# Similar proteome expression profiles of the aggregated lymphoid nodules area and Peyer’s patches in Bactrian camel

**DOI:** 10.1186/s12864-023-09715-5

**Published:** 2023-10-11

**Authors:** Yujiao Cheng, Yan Ren, Wenhui Wang, Wangdong Zhang

**Affiliations:** 1 College of Veterinary Medicine, Gansu Agricultural University, Lanzhou, Gansu China; 2 The Davies Research Centre, School of Animal and Veterinary Sciences, University of Adelaide, Roseworthy, SA 5371 Australia

**Keywords:** Bactrian camel, ALNA, PPs, Mucosal immunity, Animal domestication

## Abstract

**Background:**

The presence of Aggregated Lymphoid Nodules Area (ALNA) is a notable anatomical characteristic observed in the abomasum of Bactrian camels. This area is comprised of two separate regions, namely the Reticular Mucosal Folds Region (RMFR) and the Longitudinal Mucosal Folds Region (LMFR). The histological properties of ALNA exhibit significant similarities to those of Peyer’s patches (PPs) found in the gastrointestinal system. The functional characteristics of ALNA were examined in relation to mucosal immunity in the gastrointestinal system.

**Results:**

We used iTRAQ-based proteomic analysis on twelve Bactrian camels to measure the amount of proteins expressed in ALNA. In the experiment, we sampled the RMFR and LMFR separately from the ALNA and compared their proteomic quantification results with samples from the PPs. A total of 1253 proteins were identified, among which 39 differentially expressed proteins (DEPs) were found between RMFR and PPs, 33 DEPs were found between LMFR and PPs, and 22 DEPs were found between LMFR and RMFR. The proteins FLNA, MYH11, and HSPB1 were chosen for validation using the enzyme-linked immunosorbent assay (ELISA), and the observed expression profiles were found to be in agreement with the results obtained from the iTRAQ study. The InnateDB database was utilized to get data pertaining to immune-associated proteins in ALNA. It was observed that a significant proportion, specifically 76.6%, of these proteins were found to be associated with the same orthogroups as human immune-related genes. These proteins are acknowledged to be associated with a diverse range of functions, encompassing the uptake, processing and presentation of antigens, activation of lymphocytes, the signaling pathways of T-cell and B-cell receptors, and the control of actin polymerization.

**Conclusions:**

The experimental results suggest that there are parallels in the immune-related proteins found in ALNA and PPs. Although there are variations in the structures of LMFR and RMFR, the proteins produced in both structures exhibit a high degree of similarity and perform comparable functions in the context of mucosal immune responses.

**Supplementary Information:**

The online version contains supplementary material available at 10.1186/s12864-023-09715-5.

## Background

In addition to its role in digestion, nutritional absorption, and metabolic processes, the intestine also functions as an immunological organ, actively defending against the invasion of pathogens. The gut-associated lymphoid tissue (GALT) [1] contains around 70% of the body’s immune cells, rendering it the largest peripheral lymphoid tissue. Peyer’s patches (PPs) are an essential constituent of the GALT, distinguished by their high density of lymphatic follicles [2, 3], commonly located within the jejunum and ileum. The presence of lymphocytes causes the formation of dome-shaped elevations in the small intestine, known as Peyer’s patches (PPs). These patches consist of follicle-associated epithelium that is overlaid by the subepithelial dome, where riches B cells, T cells, and dendritic cells. The region within PPs that houses the germinal centre is referred to as the B cell region, whereas the T cell region is represented by the interfollicular space. PPs is a critical site for antigen sampling in the intestinal tract, even though it lacks lymphatic input vessels. Antigens and particles present in the gut lumen are mostly carried through microfold cells (M cells) located in the follicle-associated epithelium [4, 5]. This transportation process afterwards elicits either an adaptive immune response or immunological tolerance [6, 7]. Hence, the existence of PPs holds significance in maintaining the stability of the intestinal immune system and managing the balance between mutually advantageous symbiotic bacteria and potentially detrimental pathogenic bacteria.

The Bactrian camel, scientifically known as Camelus Bactrianus, exhibits two distinct subspecies: the domesticated Bactrian camel (*Camelus Bactrianus Linnaeus*) and the wild Bactrian camel (*Camelus Bactrianus Ferus*). These subspecies are predominantly distributed across central Asia, western China, and the high-altitude parts of India. In extremely arid desert environments, camels are highly valued as a source of meat, milk, and wool products [8]. In addition to their ability to adapt to extreme environments [9], camels possess distinct immunological characteristics, including the possession of a unique heavy chain antibody IgG devoid of a light chain [10, 11]. The morphology of the intestinal PPs in Bactrian camels exhibits polymorphism [12, 13], hence differentiating them from those seen in cattle [14, 15], horse [15],sheep [16] ,and pigs [17]. In addition to PPs, the discovery of the aggregated lymph node region (ALNA) in the abomasum in 2003 revealed a unique immune induction site in Bactrian camels [18]. The anatomical arrangement originates at the gastroesophageal junction and extends in a triangular pattern down the lesser curvature, encompassing regions characterized by reticular mucosal folds (RMFR) and longitudinal mucosal folds (LMFR) [18, 19]. A distinct demarcation exists between RMFR and LMFR, wherein the mucous folds exhibit a greater thickness compared to the surrounding tissue. The lymphoid follicles situated in the submucosal layer of the ALNA are arranged in a manner that aligns with the mucosal fissures, resulting in the formation of a well-structured mucosa-associated lymphoid tissue. Our earlier study has provided evidence that ALNA in Bactrian camels matures gradually after birth, and the process requires breast milk or antigen stimulation [20]. Following, the drop in the amount of IgA + and IgG + cells in the ageing population is observed in the ALNA [20–22]. The aforementioned properties of ALNA exhibit a congruence with intestinal PPs in cattle and sheep. Hence, an examination of the functional characteristics of ALNA in the context of digestive mucosal immunity and its parallels with PPs could potentially provide valuable insights into the distinctive immunological mechanism observed in camels.

The present work investigated the protein expression in the RMFR and LMFR individually for the ALNA, utilizing the isobaric tags for relative and absolute quantification (iTRAQ) methodology. This study aims to investigate the functional characteristics of the mucosal immunity in the digestive tract by examining the differential expression of proteins (DEPs) between PPs and ALNA. In addition, comparative genomics analyses were performed on a range of species, such as Bactrian Camel (*Camelus Bactrianus Linnaeus*), undomesticated Bactrian Camel (*Camelus Bactrianus Ferus*), Arabian Camel (*Camelus dromedarius*), alpaca (*Vicugna pacos*), cattle (*Bos taurus*), goat (*Capra hircus*), pig (*Sus scrofa*), and human (*Homo sapiens*), in order to identify orthogroups and immune-related proteins.

### Results Protein expression correlation between RMFR, LMFR and PPs

**Fig. 1:**
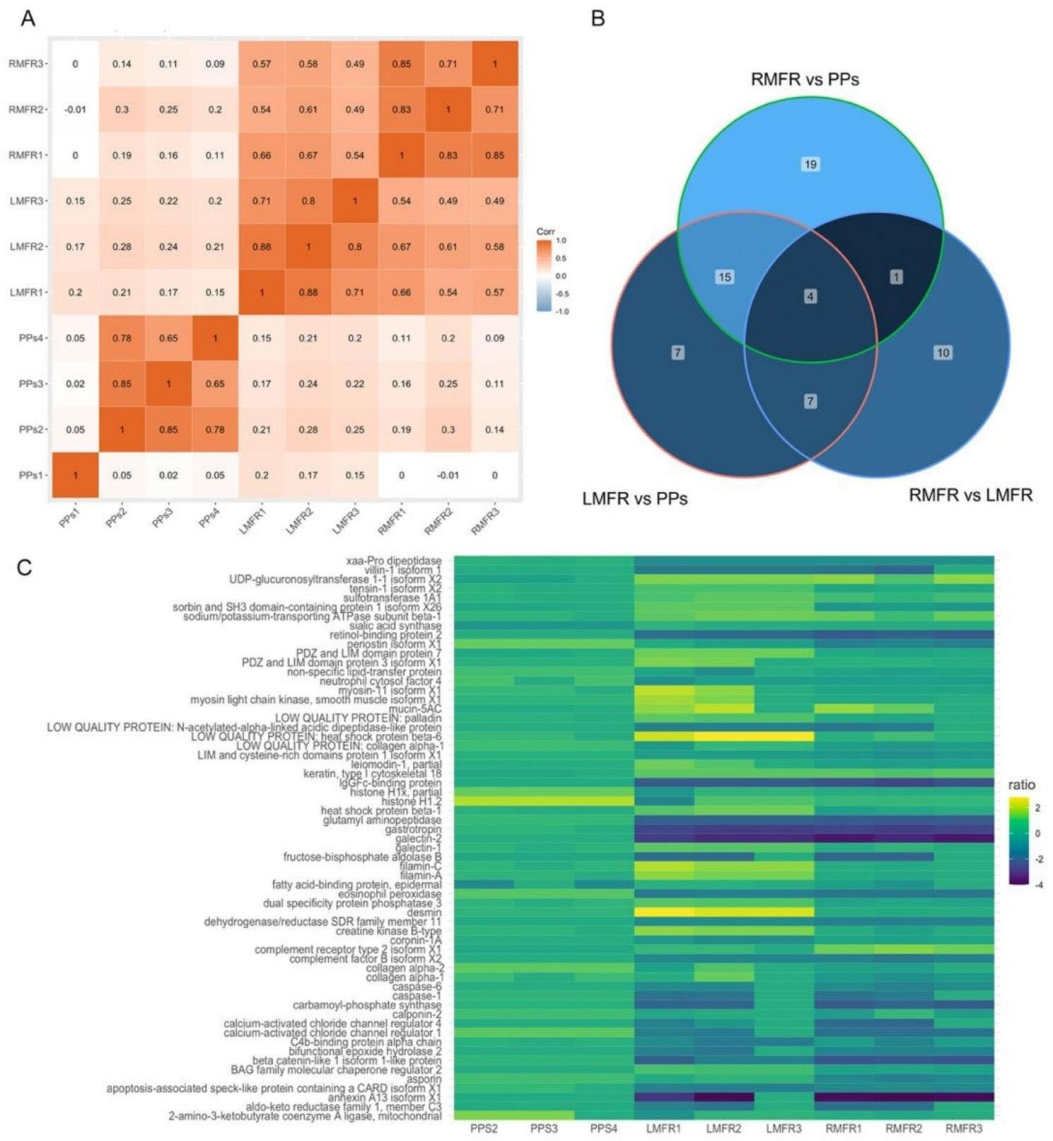
Protein expression and differential expressed protein. (**A**) Correlation of protein expression in PPs, LMFR and RMFR with 0 represents no correlation and 1 represents highest correlation. (**B**) The number of common differential expressed proteins between three comparison groups. (**C**) The reporter ions ratioin each sample for 63 common differential expressed proteins

A total of 1253 proteins have been identified in the ALNA (Table S1). Specifically, 1215 proteins were identified from the LMFR subset, whereas 1200 proteins were identified from the RMFR subset. Compared to ALNA, fewer proteins (975) were identified in PPs (Figure S1). PPs-1 exhibited a lack of success in establishing a correlation between protein expression, as it displayed a low degree of correlation with PPs-2 and PPs-3 PPs-4, as depicted in Fig. 1A. The failure of proteome analysis was confirmed for this particular sample, leading to the exclusion of PPs-1 from subsequent analyses. A strong positive correlation, with a coefficient more than 0.71, was found between LMFR-1, LMFR-2, and LMFR-3. Similarly, a significant positive correlation, also exceeding 0.71, was detected between RMFR-1, RMFR-2, and RMFR-3. In relation to the interplay between different tissues, the observed correlation coefficient between the LMFR and RMFR is greater than 0.49. Conversely, the correlation coefficients observed between the ALNA region and PPs range from 0.09 to 0.3.

### Differentially expressed proteins between RMFR, LMFR and PPs

There were 22 down-regulated proteins in RMFR when compare to LMFR. A total of 33 proteins were identified as exhibiting differential expression when comparing the proteomes of LMFR and PPs. Out of the proteins examined, a total of 13 were found to be up-regulated in LMFR, whereas 20 exhibited down-regulation in LMFR. A total of 39 proteins exhibited differential expression between RMFR and PPs, whereas four proteins shown up-regulation in RMFR and 35 proteins displayed down-regulation in RMFR (Table 1, Figure S2). There are four common differentially expressed proteins crossed all the comparisons (Fig. 1B). It is noteworthy to observe that there is a frequent occurrence of 19 DEPs that exhibit changes between PPs and both the RMFR and LMFR.

**Table 1:** Differential expressed proteins between every two tissues. There are three comparisons: RMFR vs. LMFR, RMFR vs. PPs and LMFR vs. PPs.

RMFR vs. LMFR						
Accession	logFC	FDR	Regulated	Uniprot ID	Description	Immune
XP_010972224.1	-0.30	9.1E-05	down	F2VR57	BAG family molecular chaperone regulator 2	Yes
XP_010986679.1	-0.60	2.3E-04	down	Q4R7K6	desmin	No
XP_014419228.1	-0.45	1.3E-03	down	F1SUE4	asporin	Yes
XP_006182692.1	-0.40	1.5E-03	down	F5H570	periostin isoform X1	Yes
XP_006188683.1	-0.56	1.5E-03	down	F1SEK6	beta catenin-like 1 isoform 1-like protein	Yes
XP_006174974.1	-0.56	2.0E-03	down	B7U2G6	galectin-2	Yes
XP_010947039.1	-0.26	2.4E-03	down	Q9UFN8	tensin-1 isoform X2	Yes
XP_010954554.1	-0.32	2.8E-03	down	F7G5T8	leiomodin-1, partial	Yes
XP_006190626.1	-0.25	2.8E-03	down	F1SL53	retinol-binding protein 2	Yes
XP_010958890.1	-0.26	5.5E-03	down	Q3MHW8	palladin	No
XP_010957871.1	-0.32	7.6E-03	down	A7YY43	dual specificity protein phosphatase 3	Yes
XP_006174452.2	-0.50	1.0E-02	down	F7DYX5	sorbin and SH3 domain-containing protein 1 isoform X26	No
XP_006178415.1	-0.26	2.0E-02	down	E2QXM9	PDZ and LIM domain protein 3 isoform X1	Yes
XP_010948414.1	-0.62	2.3E-02	down	F1RM62	heat shock protein beta-6	Yes
XP_010962375.1	-0.38	2.4E-02	down	F7BH02	filamin-A	Yes
XP_010969219.1	-0.34	2.4E-02	down	Q68DK3	myosin light chain kinase, smooth muscle isoform X1	Yes
XP_010958635.1	-0.26	2.6E-02	down	Q862F2	galectin-1	Yes
XP_010944493.1	-0.36	3.2E-02	down	H9F216	PDZ and LIM domain protein 7	Yes
XP_006179140.1	-0.43	3.4E-02	down	F6W6G5	myosin-11 isoform X1	Yes
XP_010945859.1	-0.43	3.8E-02	down	F1SMN5	filamin-C	Yes
XP_010970627.1	-0.39	4.0E-02	down	Q58DP7	heat shock protein beta-1	Yes
XP_010967115.1	-0.38	4.3E-02	down	G1K2D0	creatine kinase B-type	Yes
**RMFR vs. PPS**						
**Accession**	**logFC**	**FDR**	**Regulated**	**Uniprot ID**	**Description**	**Immune**
XP_006174974.1	-2.31	7.1E-09	down	B7U2G6	galectin-2	Yes
XP_006190626.1	-0.94	7.1E-09	down	F1SL53	retinol-binding protein 2	Yes
XP_010951987.1	-1.03	7.1E-09	down	F1S138	glutamyl aminopeptidase	Yes
XP_010945545.1	-1.10	7.3E-09	down	G3 × 6I0	IgGFc-binding protein	Yes
XP_006188683.1	-1.12	1.2E-08	down	F1SEK6	beta catenin-like 1 isoform 1-like protein	Yes
XP_006172811.2	-0.74	1.4E-07	down	D3Z114	Na(+)/H(+) exchange regulatory cofactor NHE-RF3 isoform 1	Yes
XP_006178035.1	-1.38	3.5E-07	down	G1NZA8	gastrotropin	Yes
XP_006181471.1	-0.77	3.5E-07	down	H2NTI5	eosinophil peroxidase	No
XP_006190219.1	-0.89	2.9E-06	down	B3KUF3	calcium-activated chloride channel regulator 1	No
XP_010958364.1	-0.97	4.3E-06	down	F1SSS0	carbamoyl-phosphate synthase	Yes
XP_006182692.1	-0.74	1.2E-05	down	F5H570	periostin isoform X1	Yes
XP_006195416.1	-0.25	1.5E-05	down	C9IYS1	LIM and cysteine-rich domains protein 1 isoform X1	Yes
XP_010959777.1	-0.44	1.6E-05	down	Q3KUS7	complement factor B isoform X2	Yes
XP_010988781.1	-0.41	1.9E-05	down	A8MXC2	dehydrogenase/reductase SDR family member 11	Yes
XP_010964927.1	-0.28	3.9E-05	down	H3BTZ5	calponin-2	No
XP_010989616.1	0.34	4.3E-05	up	E3UM64	complement receptor type 2 isoform X1	Yes
XP_014419228.1	-0.50	1.1E-04	down	F1SUE4	asporin	Yes
XP_006179793.1	-3.05	1.2E-04	down	F1RRP6	annexin A13 isoform X1	Yes
XP_010946851.1	-0.40	8.2E-04	down	E2R992	bifunctional epoxide hydrolase 2	Yes
XP_006178597.1	-0.32	1.3E-03	down	F1SFA7	collagen alpha-2(I) chain	Yes
XP_010951973.1	-0.33	1.4E-03	down	F1S131	caspase-6	Yes
XP_006182521.1	0.28	1.5E-03	up	A8D349	keratin, type I cytoskeletal 18	No
XP_010947400.1	-0.27	1.8E-03	down	Q6LAN8	LOW QUALITY PROTEIN: collagen alpha-1(I) chain	Yes
XP_010959590.1	-0.27	1.9E-03	down	F1MV98	histone H1x, partial	Yes
XP_006173738.1	-0.31	2.6E-03	down	F1RNW4	xaa-Pro dipeptidase	No
XP_006191189.1	0.46	7.2E-03	up	F7GLR5	UDP-glucuronosyltransferase 1–1 isoform X2	Yes
XP_006182239.1	-0.38	1.1E-02	down	G3U2H2	histone H1.2	No
XP_006194718.1	-0.27	1.3E-02	down	G9KCQ8	neutrophil cytosol factor 4	Yes
XP_010991737.1	-0.48	1.3E-02	down	G1SER4	caspase-1	Yes
XP_006179613.1	-0.39	1.4E-02	down	F1RQT1	LOW QUALITY PROTEIN: N-acetylated-alpha-linked acidic dipeptidase-like protein	No
XP_006186896.1	-0.40	1.4E-02	down	Q96AC8	villin-1 isoform 1	Yes
XP_014406287.1	-0.26	1.9E-02	down	F6YYK6	2-amino-3-ketobutyrate coenzyme A ligase, mitochondrial	Yes
XP_006181542.1	-0.43	2.0E-02	down	F1RIS2	apoptosis-associated speck-like protein containing a CARD isoform X1	Yes
XP_010961609.1	-0.49	2.0E-02	down	F6S6Z1	calcium-activated chloride channel regulator 4	Yes
XP_010955181.1	0.28	2.1E-02	up	H2NFZ2	mucin-5AC	Yes
XP_010996658.1	-0.28	3.1E-02	down	Q91Y61	Na(+)/H(+) exchange regulatory cofactor NHE-RF1	Yes
XP_006185294.1	-0.46	3.2E-02	down	F1SSB5	fructose-bisphosphate aldolase B	Yes
XP_006189294.1	-0.27	3.2E-02	down	F1RYI8	collagen alpha-1(III) chain	Yes
XP_006176903.1	-0.41	4.4E-02	down	G3T1J5	aldo-keto reductase family 1, member C3	No
**LMFR vs. PPS**						
**Accession**	**logFC**	**FDR**	**Regulated**	**Uniprot ID**	**Description**	**Immune**
XP_010951987.1	-0.88	2.6E-06	down	F1S138	glutamyl aminopeptidase	Yes
XP_006174974.1	-1.75	4.0E-06	down	B7U2G6	galectin-2	Yes
XP_010945545.1	-0.88	5.3E-06	down	G3 × 6I0	IgGFc-binding protein	Yes
XP_006178035.1	-1.46	1.3E-05	down	G1NZA8	gastrotropin	Yes
XP_006190626.1	-0.69	1.3E-05	down	F1SL53	retinol-binding protein 2	Yes
XP_006182692.1	-0.34	3.5E-05	down	F5H570	periostin isoform X1	Yes
XP_006172811.2	-0.59	5.5E-05	down	D3Z114	Na(+)/H(+) exchange regulatory cofactor NHE-RF3 isoform 1	Yes
XP_006181471.1	-0.66	8.0E-05	down	H2NTI5	eosinophil peroxidase	No
XP_006187837.1	0.26	9.5E-05	up	Q5TBR1	sialic acid synthase	Yes
XP_010986679.1	0.54	1.2E-04	up	Q4R7K6	desmin	No
XP_006188683.1	-0.55	6.5E-04	down	F1SEK6	beta catenin-like 1 isoform 1-like protein	Yes
XP_010959777.1	-0.35	1.7E-03	down	Q3KUS7	complement factor B isoform X2	Yes
XP_006190219.1	-0.56	3.3E-03	down	B3KUF3	calcium-activated chloride channel regulator 1	No
XP_010988781.1	-0.26	3.6E-03	down	A8MXC2	dehydrogenase/reductase SDR family member 11	Yes
XP_006183131.1	0.25	5.7E-03	up	E9PKR8	sulfotransferase 1A1	Yes
XP_006184383.1	-0.27	5.7E-03	down	E2QSZ5	coronin-1 A	Yes
XP_010996658.1	-0.53	8.7E-03	down	Q91Y61	Na(+)/H(+) exchange regulatory cofactor NHE-RF1	Yes
XP_010980422.1	-0.28	9.7E-03	down	F7C7P2	non-specific lipid-transfer protein	Yes
XP_010958364.1	-0.57	1.2E-02	down	F1SSS0	carbamoyl-phosphate synthase	Yes
XP_006174452.2	0.32	1.3E-02	up	F7DYX5	sorbin and SH3 domain-containing protein 1 isoform X26	No
XP_006182521.1	0.32	1.3E-02	up	A8D349	keratin, type I cytoskeletal 18	No
XP_006179793.1	-2.08	1.7E-02	down	F1RRP6	annexin A13 isoform X1	Yes
XP_010948414.1	0.58	1.9E-02	up	F1RM62	heat shock protein beta-6	Yes
XP_010944493.1	0.37	2.0E-02	up	H9F216	PDZ and LIM domain protein 7	Yes
XP_006173308.1	0.27	2.2E-02	up	Q58I20	sodium/potassium-transporting ATPase subunit beta-1	Yes
XP_010946851.1	-0.34	2.7E-02	down	E2R992	bifunctional epoxide hydrolase 2	Yes
XP_010962375.1	0.31	3.3E-02	up	F7BH02	filamin-A	Yes
XP_014422033.1	-0.47	3.6E-02	down	F1S0J2	C4b-binding protein alpha chain	Yes
XP_010945859.1	0.39	3.8E-02	up	F1SMN5	filamin-C	Yes
XP_006193989.1	0.30	4.1E-02	up	Q30B88	fatty acid-binding protein, epidermal	Yes
XP_006186896.1	-0.48	4.6E-02	down	Q96AC8	villin-1 isoform 1	Yes
XP_006191189.1	0.46	4.7E-02	up	F7GLR5	UDP-glucuronosyltransferase 1–1 isoform X2	Yes
XP_010967115.1	0.33	4.8E-02	up	G1K2D0	creatine kinase B-type	Yes

Using a heatmap, the expression levels of the 62 differently expressed proteins in the pie chart were further visualized (Fig. 1C). Compared to the RMFR, proteins in the LMFR have a high level of expression, and most of these proteins (86.4%) are associated with immunological functions (Table 1). Eight proteins, including BAG2, PALLD, DUSP3, SORBS1 isoform X26, PDLIM3 isoform X1, FLNA, MYH11 isoform X1, and FLNC are related to actin binding (Viewed in UniProt Sep, 2023) [23]. Besides, MYH11 and myosin light chain kinase, smooth muscle isoform X1 proteins are playing important roles in ATP generation and myosin II complex assembly [24].

The actin-myosin filament interaction is recognized for enabling myosin to operate as a motor, propelling the motion of actin filaments and facilitating muscle contraction [25, 26]. DEPs related to platelet-derived growth factor (e.g. COL1A1 and COL1A2), immunoglobulin receptor (e.g. FLNA and CR2) or immunoglobulin receptor binding (e.g. SCP2 and GCAT) can be observed between ALNA and PPs.

### Biological pathway identification and enrichment analysis

#### Biological pathway identification and enrichment for ALNA region

**Fig. 2:**
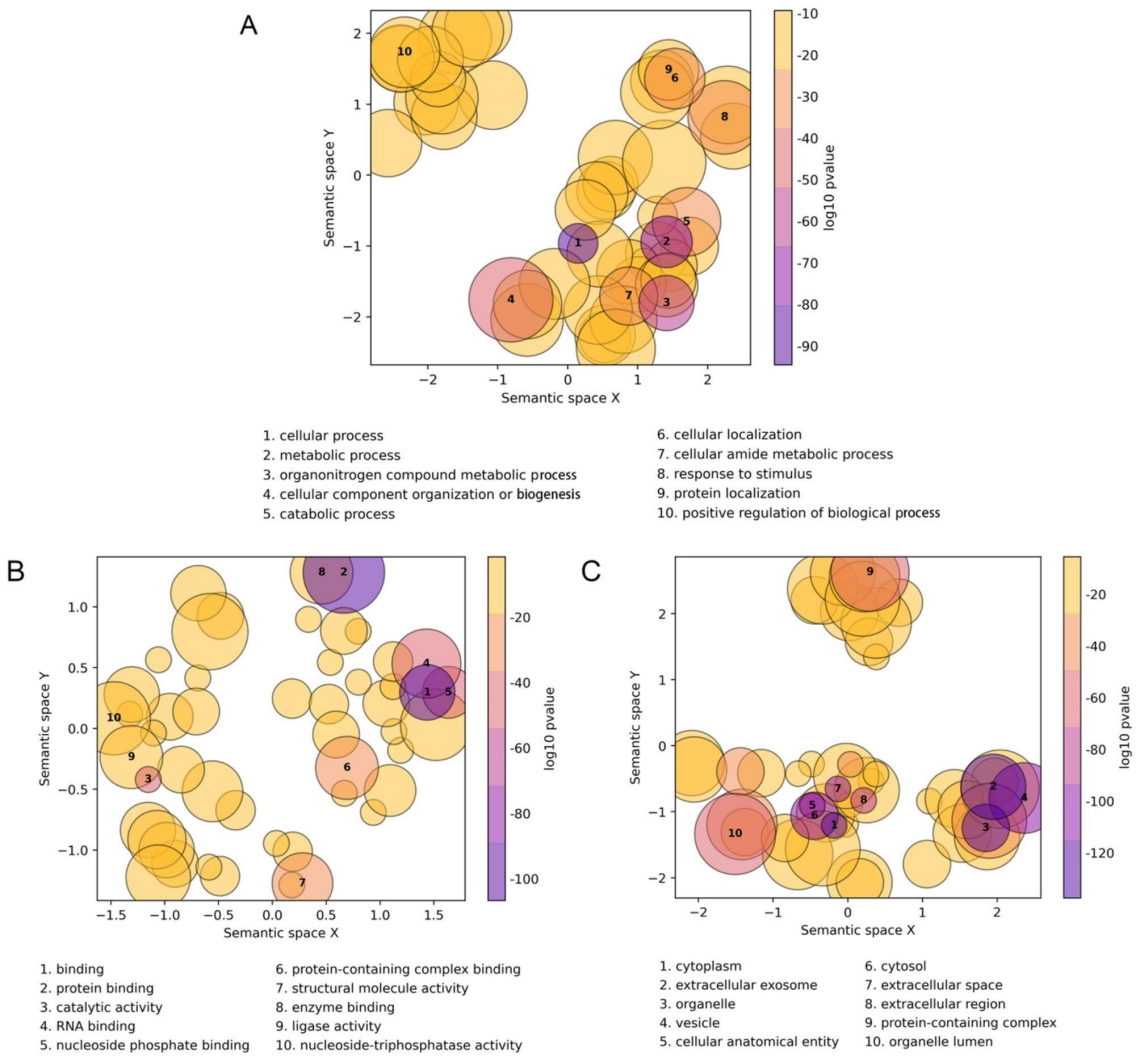
Enriched GO terms on proteins expressed in ALNA region. (**A**) Enriched GO terms of biological process. (**B**) Enriched GO terms of molecular function. (**C**) Enriched GO terms of cellular component

In order to enhance comprehension of the biological principles pertaining to the ALNA region, an analysis of Gene Ontology (GO) enrichment was conducted on the proteins expressed within this region. The enriched GO terms were organized into 345 clusters. The descriptions of the highest ranking clusters are presented in Fig. 2, categorized according to biological process (Fig. 2A), molecular function (Fig. 2B), and cellular component (Fig. 2C). Each circular representation corresponds to a distinct cluster of GO terms that have a common functional attribute. The size of each circle is indicative of the number of GO terms encompassed within the respective cluster. The color represented by the logarithmically transformed P-values serves as an indicator of the statistical importance of the GO cluster.

It is evident that the enriched clusters exhibit associations with metabolic activities, energy production, and substance secretion. To provide further clarification, the analysis revealed the identification of the organonitrogen compound metabolic process (GO:1,901,564), the cellular amide metabolic process (GO:0043603), cellular component organization or biogenesis (GO:0071840), and structural molecule activity (GO:0005198). The result demonstrates that ALNA actively participates in metabolic and material conversion processes. Due to close to the pyloric gland of the abomasum, ALNA is impossible to disregard its complex physiological environment with low pH chemical stimulation and absorption and metabolism of substances.

#### Biological pathway enrichment analysis of differentially expressed proteins

The GO and Kyoto Encyclopedia of Genes and Genomes (KEGG) enrichment analysis were done for each group of differentially expressed proteins (Table S2, Table S3). Fifty-three GO terms were identified as significantly enriched (false discovery rate, FDR < 0.05) for differentially expressed proteins between RMFR and PPs. Terms including cellular response to amino acid stimulus (GO:0071230), collagen fibril organization (GO:0030199), extracellular matrix structural constituent conferring tensile strength (GO:0030020), and collagen type I trimer (GO:0005584) are at the end of the directed acyclic graph (DAG) virilized by QuickGO (https://www.ebi.ac.uk/QuickGO/). KEGG didn’t reveal too many informative terms except protein digestion and absorption (hsa04974).

The differentially expressed proteins between LMFR and PPs can be enriched into 43 GO terms and 1 KEGG terms. Terms regulation of monoatomic cation transmembrane transport (GO1904062), positive regulation of monoatomic ion transmembrane transport (GO:0034767), extracellular exosome (GO:0070062), fatty acid binding (GO:0005504) and actin filament bundle (GO:0032432) are listed at the bottom of DAG. The KEGG pathways reviewed the term MAPK signaling pathway (hsa04010). In summary, the significant pathways responsible for the synthesis and activation of biological enzymes, as well as amino acid metabolism, are observed to occur between the ALNA and PPs. The observed phenomenon can be attributed to variances in the physiological conditions of the stomach and metabolic absorption, resulting in distinct microenvironments and immunological functionalities.

There were 38 GO and 1 KEGG terms enriched for differentially expressed proteins between LMFR and RMFR. Terms including stress fiber (GO:0001725) and Z disc (GO:0030018) myofibril assembly (GO:0030239) and galectin complex (GO:1,990,724) are on the bottom of DAG. The KEGG enrichment analysis yielded significant results indicating the presence of the Mitogen-Activated Protein Kinase (MAPK) signaling pathway (hsa04010). There exist pivotal signaling pathways that govern a diverse array of cellular functions, encompassing proliferation, differentiation, apoptosis, and stress responses [27]. The aforementioned pathways are accountable for cellular motility and cytoskeletal reinforcement, activation of protein phosphorylation, as well as metabolism and transportation of substances.

### Cross species comparative genomics analysis

**Fig. 3:**
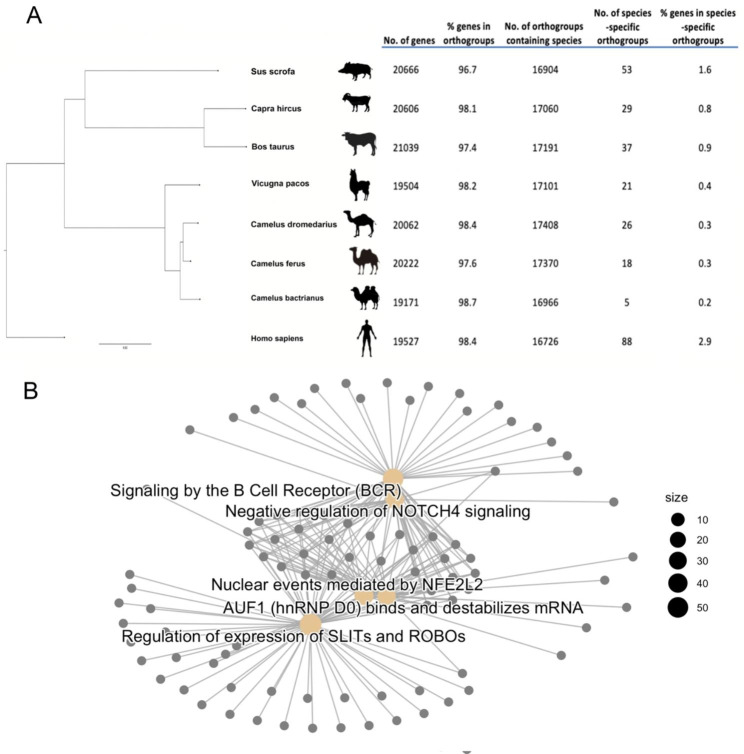
Cross species comparative genomics statistic and pathways of immune-related proteins in ALNA region. (**A**) Phylogenetic tree and orthogroup statics of eight selected species. (**B**) Enriched pathway of immune-related protein identified in ALNA region

A phylogenetic tree of domesticated Bactrian camel, undomesticated Bactrian camel, Arabian camel, alpaca, cattle, goat, pig and human was shown in the Fig. 3A. The phylogenetic distance between two species estimates the amount of time since the most recent common ancestor of both species [28]. Compared to other selected species, the divergence time between Arabian camel and undomesticated Bactrian camel is the shortest on the phylogenetic tree. Domesticated Bactrian camel diverged earlier than Arabian camel. Alpaca from Camelidae family is in the same cluster as three Camelids species, whereas cattle, goat, pig are in another cluster sharing a common ancestor. Humans are the most divergent species among selected species.

In total of 18,426 orthologous groups were identified among eight species (Table S4). To study the genetic influence of domesticate activities in camel, we studied the orthologous groups that potentially causes the genetic difference between domesticated Bactrian camel and other two camel subspecies. More gene copies were found in domesticated Bactrian camel for three gene families, protocadherin gamma subfamily C, CD179 antigen-like family member A and vomeronasal type-1 receptor 1 (Table 2, group 1). Among them, vomeronasal type-1 receptor 1 is related to olfactory receptor [29]. Family protocadherin gamma subfamily C is correlated to protocadherin production. The family CD179 antigen-like family member A is interact with protocadherin fat 1 [30]. More interestingly, researchers found that cooperation between olfactory receptor genes and protocadherin genes is correlated with obesity in human [31]. We found fewer gene copies in domesticated Bactrian camel for melanin-correlated gene families such as melanoma-associated antigen, melanocortin-2 receptor accessory protein 2-like, and trafficking protein particle complex subunit 9-like (Table 2, group 2). Less copies have also been found for family ankyrin repeat and olfactory receptor 7 in domesticated Bactrian camel. Two structural constituents of ribosome gene families, the ribosomal L29e protein family and ribosomal protein L32 gene family have been found with more gene copies in undomesticated Bactrian camel (Table 2, group 3). Another family found with more gene copies in undomesticated Bactrian camel is ferritin light chain. The ferritin light chain mRNA is a cap structure that recruits small ribosomal subunits to the mRNA for ferritin synthesis [32]. There are fewer copies of the gene family’s olfactory receptor family 6 and protocadherin alpha in Arabian camels.

**Table 2:** Orthologous groups of gene families with different number of gene copies among domesticated Bactrian, Bactrian camel and Arabian camel. The Type column marked for different conditions: The gene families that domesticated Bactrian contain more gene copies than the others marked as type 1; The gene families that domesticated Bactrian contain less gene copies than the others marked as type 2; The gene families that undomesticated Bactrian contain the most gene copies marked as type 3; The gene families that Arabian camel contains the lest gene copies marked as type 4

Family name	domesticated Bactrian camel	Arabian camel	undomesticated Bactrian camel	Group
protocadherin gamma subfamily C	10	2	3	1
CD179 antigen-like family member A	11	1	1	1
vomeronasal type-1 receptor 1	7	0	0	1
melanoma associated antigen family	4	11	10	2
ankyrin repeat family	7	21	25	2
olfactory receptor family 7 subfamily A	1	7	6	2
trafficking protein particle complex subunit 9-like	0	4	8	2
melanocortin-2 receptor accessory protein 2-like	0	7	4	2
ribosomal L29e protein family	4	5	14	3
ribosomal protein L32 gene family.	1	3	9	3
ferritin light chain	2	2	17	3
olfactory receptor family 6	22	13	18	4
protocadherin alpha	10	5	8	4

### Immune-related gene prediction

By using the human InnateDB database, we identified 731 immune-related proteins in the ALNA region of domesticated Bactrian camel (Table S5). The genes responsible for encoding these proteins are categorised within the same ortholog groupings as the well-established immune genes found in humans. Consequently, a total of 566 proteins associated with immune responses were identified for the purpose of conducting pathway enrichment analysis. These proteins exhibited significant enrichment in 283 pathways (Table S6). The analysis of the top five terms provides insights that lead us to three distinct directions (Fig. 3B). Pathway AUF1 (hnRNP D0) binds and destabilizes mRNA and Nuclear event mediate by NFE2L2 are in the same cluster, which correlates to heat stress response. Similar terms, such as Regulation of HSF1-mediated heat shock response (HSR) (Q-value: 1.33E-05) also could be identified with significance (Table S6). Another direction including terms signalling by the B cell receptor and negative regulation of NOTCH4 signalling, which implies the role of ALNA region in immune response. Other terms that point to this direction are Downstream signalling events of B Cell Receptor (Q-value: 2.35E-10), Activation of NF-kappaB in B cells (Q-value: 1.34E-09), Antigen activates B Cell Receptor leading to generation of second messengers (Q-value: 2.10E-02), Signalling by NOTCH (Q-value: 1.13E-03), Antigen Presentation: Folding, assembly and peptide loading of class I MHC (Q-value: 2.88E-03), Downstream TCR signaling (Q-value: 4.22E-08), CLEC7A (Dectin-1) signaling (Q-value: 7.42E-08)and C-Type Lectin Receptors (Q-value: 7.61E-06). The last direction represents by the regulation of expression of SLITs and ROBOs, which usually regulates physiological cell functions in adult reproductive tissues.

The comparison of tissues revealed that there were 31 immune-related proteins that exhibited differential expression between RMFR and PPs. A total of 28 immune-related proteins have been identified as exhibiting differential expression between the LMFR and PPs. A total of 19 proteins exhibiting differential expression between LMFR and RMFR have been found to be associated with immunological functions (Table 1). The comparison groups were subjected to pathway enrichment analysis using the human InnateDB database (Table S7, Figure S3). The lack of an adequate number of proteins resulted in unclear clusters of phrases. However, it was still possible to identify terms such as smooth muscle constriction and muscular constriction from the DEPs between RMFR and LMFR.

### Validation of DEPs from three comparison groups using ELISA

In order to verify the statistical findings obtained from the iTRAQ data, the essential DEPs FLNA, MYH1, and HSPB1 were subjected to validation using ELISA. Proteins are known to fulfil crucial functions in various biological processes, including structural organization, regulation of cellular motility, and stabilization of the cytoskeleton. The ELISA results demonstrated a consistent expression pattern of these three proteins, which aligns with the findings obtained from the iTRAQ-based proteomic data (Figure S4).

## Discussion

This is the first study to quantify the protein expression and immune-related proteins in the ALNA. Compared to PPs, more proteins can be identified in the ALNA. Those proteins are involved in purine nucleotide binding and pyrophosphatase. The exosomes produced by immune cells in this region will be secreted into the extracellular environment for chemical and organic stimulations.

As a result of studying the immune-related proteins in ALNA, we identified pathway terms are cluster for heat stress response, mucosal immune response, and physiological cell regulation. The follicular zone, and parafollicular zone in the SED were discovered to be abundant in macrophages, DC, T cells, and B cells [18, 20] in the earlier study on the histological characteristics of ALNA, indicating the immunological response of ALNA to antigen. A variety of mucosal immune-related proteins have been identified expressed in ALNA, which majorly point to antigen uptake, processing, and presentation; activation of lymphocytes; and the functioning of MHC I and MHC II. Such as Downstream TCR signalling, Activation of NF-kappaB in B cells, Antigen activates B Cell Receptor leading to generation of second messengers, Folding, assembly and peptide loading of class I MHC. C-Type Lectin Receptors(CLRs)and CLEC7A (Dectin-1) signalling were also enriched. CLR is a pattern recognition receptor found on the outer membrane of phagocytic cells. These receptors play a crucial role in the identification of pathogens, the process of phagocytosis, and the transmission of inflammatory signals [33]. CLRs are one of the innate immune receptors that facilitate the transition from innate to adaptive immune responses through mediating cell-to-cell contacts [34, 35]. The gastrointestinal immune system faces the challenge of maintaining a balance between accepting symbiotic bacteria and dietary antigens while defending against harmful pathogens due to the complex nature of the gastrointestinal environment. The family of CLR proteins provides the necessary conditions for this immunological homeostasis. One specific member of this family, Dectin-1, has been demonstrated to regulate the differentiation and demise of T cell populations as well as the composition of the gastrointestinal microbiome, thereby contributing to the maintenance of immune homeostasis [36, 37]. Furthermore, as previously stated, PPs primarily depend on M cells for the facilitation of material transportation. The transportation of secretory IgA through M cells in PPs is contingent upon the existence of Dectin-1 and Siglece − 5 receptors [38] The findings of our study indicate that there is no significant difference in the proteins of the CLRs-related pathway between ALNA and PPs. This suggests that these proteins likely have similar biological functions in the two different tissues. Signaling by NOTCH can be involved in T cell maturation and differentiation [39, 40]. Intraepithelial lymphocytes (IELs) are a group of cells in the epithelium of the mucous membrane of the small intestine that are mostly made up of T cells IELs, as opposed to other T cells, originate and mature in the intestines [41, 42]. Further study is necessary to ascertain whether the notch pathway revealed in ALNA enhances IEL maturation in the abomasum of Bactrian camels. In the gut, NOTCH signalling also determines the maintenance, proliferation, and differentiation of intestinal stem cells [43]. It coordinates the lineage commitment of differentiated cells, including secretory cell lineages and absorptive cell lineages, to maintain environmental stability in the intestine [44]. It is potential that the Notch receptor and other proteins play equally significant functions in ALNA. ALNA and PPs are both highly organized mucosal lymphatic tissues of the gastrointestinal tract, but their locations differ. Our research indicates that DEPs of ALNA and PPs are not directly implicated in these immune-related pathways. Therefore, we conclude that there is no significant difference between ALNA and PPs in these immune functions.

Camels have evolved unique physiological and biochemical characteristics in order to cope with the extreme heat and dehydration of the desert. It has been proposed that the HSR plays an important function in protein homeostasis and thermotolerance [45]. HSF1-mediated HSR transcription is activated by heat shock factor protein 1, which produces a large class of molecular chaperones, heat shock proteins [46]. These proteins belong to the HSPBA, HSPB, and HSPC protein families. The HSP70 family, also referred to as HSPBA, comprises proteins that are characterized by their high conservation and a molecular weight of 70 kDa. The proteins are chiefly acknowledged for their capacity to endure high temperatures and mitigate stress. The ALNA expresses two HSPBA proteins, including HSPA8 and HSPA5. The HSPB is a group of low molecular weight heat shock proteins with a weight less than 43 kDa. Their influence on cellular resistance to stress is noteworthy. The expression of MAPK1 and MAPK3 was observed in ALNA. MAPK catalysed phosphorylation sites have the potential to enhance the cytoprotective and chaperone functions of HSPB5 (αB-crystallin, CRYAB) in terms of stress relief, induced protein aggregation and stabilization [47]. Meanwhile, the findings suggest that the expression of HSPB1 in LMFR is in line with the protein trend of actin filaments, indicating the significant contribution of small heat shock protein chaperones in actin polymerization and maintenance of cytoskeletal stability [48]. The molecular weight of HSPC (HSP90 Family) constituents has been approximated to be 90 kDa, and they exhibit broad expression across various cell and tissue categories [49]. Proteins HSP90AA1 and HSP90AB1 expressed in ALNA play a key role in maintaining vital physiological processes and many pathologies [50–53].

In our previous study, we found the bacterial communities to be similar between the RMFR and LMFR [54]. The similar bacterial structure suggests that these differences may not be caused by bacterial parasitism, but rather to the host’s internal expression, which implies distinct physiological patterns for RMFR and LMFR. Myosin-related proteins, for example, are more abundant in LMFR than in other tissues. Myosin is a protein that converts ATP into mechanical energy, which is responsible for many types of cell movement [26]. The migration of immune cells from mucosal inductive sites to effector sites through the lymphatic system constitutes the cellular foundation of the immune response [55]. Another example is that more actin binding proteins are expressed in LMFR than RMFR. Actin can interact with myosin filaments and contribute to smooth muscle contraction [25, 26], which could be an explanation of the different textures between LMFR and RMFR. According to previous findings, less FcRn expressed in LMFR for camel of pubertal age. Vascular smooth muscle constriction could be a clue to explain the lower level of FcRn expression in LMFR compared to RMFR [56]. This paper further supports the idea that FcRn transmembrane transport involving IgG could participate in mucosal immune responses in ALNA. Furthermore, both B cell and T cell receptor signaling pathways were turned up for DEPs between RMFR and LMFR. B cells and T cells are predominantly found in the lamina propria, where FcRn was also differentially expressed between RMFR and LMFR, suggesting that FcRn transmembrane transport involving IgG could contribute to the mucosal immune response in ALNA [56]. Moreover, we compared the protein expressions between the ALNA region and PPs and found a significant difference, although they are both mucosal immune inductive sites of the gastrointestinal tract. Since we could make a suspicion that different bacteria colonized in ALNA and ileal PPs of Bactrian camel could be a cause for the difference in protein expression, which can be accomplished through host-microbe protein-protein interactions [57].

Cross-species comparative genomics analysis was running for two reasons. To begin, use genes from the same orthogroups as human genes to identify immune-related proteins. Second, because a hybrid of domesticated and wild Bactrian camels exists, we are keen to know the impact of domestication on Bactrian camels. As a result, it is interesting to see fewer melanoma gene copies in domestic Bactrian camels. Domestic Bactrian camels were raised with half-year pasture gazing and half-year feedlot feeding, which mean they have less chance exposed to the sun-exposed environments. This indicates domestication of camels may weaken their ability of heat adaption. Besides, gene copies of olfactory receptor families are often found various between domestic and non-domestic animals. Despite camels, similar evidence has been reported in cattle with the expansion of sperm olfactory receptor genes in domesticated cattle, which could have important implications for understanding selection for male fertility [58]. There is no significant expansion of olfactory receptor genes in domesticated Bactrian camels compared to non-domesticated camels. However, further study needs to be done using sperm tissues.

Despite the tremendous findings, limitations and potential directions also need attention. We resort to the human database because our current arsenal of camel tools and databases relating to immune-related proteins is insufficient. On the phylogenetic tree, however, the human is the most diverse species among the selected species. Camel and its close relatives’ species-specific proteins cannot be included in our study. Although our study has limitations, it still provides clues for better understanding the ALNA region. Provide direction for future studies on the biological functions of ALNA in mucosal immune and stress responses.

## Conclusions

This study presents a comprehensive overview of the protein expression patterns in ALNA and performs a comparative examination of the DEPs between ALNA and PPs. In addition, we performed a pioneering cross-species investigation by employing the human immune gene repository to find immune proteins belonging to the same orthogroups. The results suggest that there are parallels between the immune-related proteins identified in ALNA and PPs. This study contributes to our understanding of ALNA and offers support for further exploration of gastrointestinal mucosal immunity in camels.

## Materials and methods

### Animal samples

The animals used in this study were sourced from Minqin County, Gansu Province, China. Twelve healthy pubertal Alashan Bactrian camels from ages 3 to 5 were randomly selected among a few grazing camels. The selected animals were anaesthetized intravenously with sodium pentobarbital (20 mg/kg) and exsanguinated until death. The ileum PPs, LMFR, and RMFR of the abomasum were taken from each Bactrian. A total of 30 tissue samples were collected. In order to mitigate the impact of individual variations, a practice was implemented wherein tissues from three animals were combined as a collective group and afterwards identified under a single label. As a result, the participants were categorized into six distinct groups, namely PPs-1 (failed), PPs-2, PPs-3, PPs-4, LMFR-1, LMFR-2, LMFR-3, RMFR-1, and RMFR-2, RMFR-3.

### Protein sample preparation and iTRAQ labeling

Tissue samples were pulverized in liquid nitrogen, then extracted by lysis buffer (7 M urea, 2 M thiourea, 0.2%SDS, 40 mM Tris-HCl, pH 8.5) containing 1 mM PMSF and 2 mM EDTA (final concentration). After sonication and centrifugation, the supernatant was combined with a 5-fold volume of cold acetone that was incubated overnight at -20℃. The precipitate was then washed three times with cold acetone, and the pellets were air-dried. The extracted proteins were examined by Bradford assay and SDS-PAGE Coomassie staining (Figure S5).

For each sample, prepare 100 g protein solution, add 5 g Trypsin Gold (Promega, Madison, WI, USA), and enzymolize at 37 °C overnight. To remove salt from the hydrolyzed peptide and vacuum drain, a Strata X C18 column was employed. 0.5 M TEAB polypeptide was added, and six iTRAQ labelings were carried out in accordance with the manual (Applied Biosystems, FosterCity, CA, USA). ITRAQ reagents were used to label the proteins in each tissue as follows: PPs-1(113) PPs-2(117); LMFR-1(114) LMFR-2(119); RMFR-1(116) RMFR-2(121). An additional four samples were labelled and included in the examination conducted in batch 2: PPs-3(116), PPs-4(117), RMFR-3(118) and LMFR-3(119). The batch effect was controlled using the method described in Jelena’s paper [59], and the distance and correlation between distinct batches were evaluated separately prior to and after batch correction (Figure S6).

### Separation of peptides and LC-MS/MS analysis

The labeled samples were combined and purified with Gemini C18 column (Phenomenex, Torrance, CA, USA), and separated with LC-20AB HPLC system (Shimadzu, Kyoto, Japan). Absorbance at 214 nm was applied to monitor elution, and fractions were collected every minute. Collected fraction was reconstituted by Buffer A (2%ACN, 0.1%FA) and centrifuged at 20,000 g for 10 min. The supernatant was injected into an LC-20AD and separated using liquid chromatography. The peptides were subsequently eluted and separated to use a 10 cm analytical C18 column (inner diameter 75 m) in gradient buffer B (98%ACN,0.1% FA).

The data was collected with a Triple TOF5600 System (SCIEX, Framingham, USA) in conjunction with a Nanospray III source (SCIEX, Framingham, USA) and a drawn quartz tip as the emitter (New Objectives, USA). And the ion spray voltage at 2.3 kV, the curtain gas pressure at 30psi, the nebulizer gas pressure at 15psi, and the interface heater temperature at 150 °C. The labelling efficiency is 90%, and the distribution of the peptide length was shown in Figure S7.

### Bioinformatics iTRAQ data analysis

The SearchGUI v4.0.27 [60] for configuring the freely available open mass spectrometry search algorithm (OMSSA) and X!Tandem search engines were used to search for the proteins. Bactrian camel reference databases BCGSAC_Cfer_1.0 (GCA_009834535.1), CB1 (GCA_000604445.1) and Ca_bactrianus_MBC_1.0(GCA_000767855.1)are combined for searching to cover more known proteins (Table S8). There were 125,932 annotated proteins in the combined database. After removing duplications, 83,120 annotated proteins have been used by the search engines. The parameters for searching are shown in Table S9. The results of identified peptides are introduced to PeptideShaker v1.16.45 [61] for filtering and selection. There were 1253 proteins originally identified by setting at least one peptide covering the protein with > 95% reliability. The ion intensity values were log2 transformed and underwent normalization using the trimmed mean of M-values approach (TMM). Subsequently, a linear model was applied to identify differentially expressed proteins. The results were subjected to filtering based on a FDR threshold of less than 0.05 and a minimum log fold change (LogFC) of 0.5 for identifying statistically relevant targets.

### Biological pathways analysis

The GO and KEGG pathway analysis were conducted for different tissues and the lists of DEPs. The GO enrichment analysis was conducted on the Geneontology website (http://geneontology.org), and the resulting summary visualizations were generated using the GO-Figure tool [62]. The Reactome platform is utilized to perform pathway enrichment analysis on genes associated with the immune system.

### Cross species orthologous group and immune gene identification

A comparative genomics analysis was conducted to compare a few camel species and other close livestock, along with human as the control. The analysis included domesticated Bactrian camel (*Camelus Bactrianus Linnaeus*), undomesticated Bactrian camel (*Camelus Bactrianus Ferus*), Arabian camel (*Camelus dromedarius*), alpaca (*Vicugna pacos*), cattle (*Bos taurus*), goat *(Capra hircus)*, pig *(Sus scrofa)* and human (*Homo sapiens*). By suing the tool OrthoFinder, we identified orthologous groups among those species. We calculate the number of different genes for each orthologous group with the formula abs((Genes_Cdro_+ Genes_Cfer_)/2 - Genes_Cbac_), and ranked the differences.

### Immune-related gene identification

The orthologous group of camel genes was examined to identify human genes that have similarities. Then, human genes were employed for querying the InnateDB [63] in order to identify genes associated with the immune system. The most recent InnateDB has a total of 6714 genes that are associated with the immune system. Every gene is associated with a distinct Entrez gene identifier, which can be linked to the UniportKB identifiers of the relevant proteins.

### Protein validation with ELISA

Tissue PPs, LMFR, and RMFR were accurately weighed for 0.5 g, and three biological replicates were obtained for each sample. Adding 1 ml of PBS and two magnetic beads, homogenize for 15 min at -10 °C before centrifuging for 10 min at 4 °C at 12,000 rpm. Retrieve the supernatant. The protein content was measured using the BCA protein Assay Kit (Solarbio, Beijing, China). The expression levels of filamin-A (FLNA), heat shock protein beta 1 (HSPB1), and myosin-11 (MYH11) were assessed in various groups using ELISA kits (FLNA ELISA Kit, YJ955247, Shanghai Enzyme Linked Biotechnology, China; HSPB1 ELISA Kit, YJ514796, Shanghai Enzyme Linked Biotechnology, China; MYH11 ELISA Kit, YJ981540, Shanghai Enzyme Linked Biotechnology, China). All experimental results were represented as the mean ± standard error of mean (SEM). A one-way analysis of variance (ANOVA) was performed using GraphPad Prism 9.5 (GraphPad, Inc., San Diego, CA, USA) to examine the differences between the three groups.

### Electronic supplementary material

Below is the link to the electronic supplementary material.

Supplementary Material 1

## Data Availability

All mass spectrometry data in this paper are available in the platform iProX (Project ID: IPX0006317000). https://www.iprox.cn/page/project.html?id=IPX0006317000.

## Code Availability

The code to perform various protein expression studies is available on Github (https://github.com/KellyDREN/Camel_ALNA_Protein_Quantification_paper).

## Abbreviations

ALNA: Aggregated lymphoid nodules area
RMFR: reticular mucosal folds region
LMFR: longitudinal mucosal folds region
PPs: Peyer’s patches
differentially expressed proteins: DEPs
GALT: gut-associated lymphoid tissue
M cells: microfold cells
CLRs: C-Type Lectin Receptors
IEL: intraepithelial lymphocyte
HSR: heat shock response
FDR: false discovery rate
DAG: directed acyclic graph

## References

[1] Neutra MR, Mantis NJ, Kraehenbuhl JP (2001). Collaboration of epithelial cells with organized mucosal lymphoid tissues. Nat Immunol.

[2] Jung C, Hugot JP, Barreau F (2010). Peyer’s patches: the Immune Sensors of the intestine. Int J Inflam.

[3] Randall TD, Carragher DM, Rangel-Moreno J (2008). Development of secondary lymphoid organs. Annu Rev Immunol.

[4] Ohno H (2016). Intestinal M cells. J Biochem.

[5] Man AL, Prieto-Garcia ME, Nicoletti C (2004). Improving M cell mediated transport across mucosal barriers: do certain bacteria hold the keys?. Immunology.

[6] De Obaldia ME, Bhandoola A (2015). Transcriptional regulation of innate and adaptive lymphocyte lineages. Annu Rev Immunol.

[7] Reboldi A, Cyster JG (2016). Peyer’s patches: organizing B-cell responses at the intestinal frontier. Immunol Rev.

[8] Zongping L (2003). Studies on the haematology and trace element status of adult bactrian camels (Camelus bactrianus) in China. Vet Res Commun.

[9] Wu H, Guang X, Al-Fageeh MB, Cao J, Pan S, Zhou H (2014). Camelid genomes reveal evolution and adaptation to desert environments. Nat Commun.

[10] Hamers-Casterman C, Atarhouch T, Muyldermans S, Robinson G, Hamers C, Songa EB (1993). Naturally occurring antibodies devoid of light chains. Nature.

[11] Nguyen VK, Hamers R, Wyns L, Muyldermans S (2000). Camel heavy-chain antibodies: diverse germline V(H)H and specific mechanisms enlarge the antigen-binding repertoire. EMBO J.

[12] Qi SS, Wang WH, Gao Q, Xu XH, He WH, Zhaxi YP (2011). Age-related changes in the anatomical characteristics of Peyer’s patches in small intestine of bactrian camels (Camelus bactrianus). Trop Anim Health Prod.

[13] ZhaXi Y, Wang W, Zhang W, Gao Q, Guo M, Jia S (2014). Morphologic observation of mucosa-associated lymphoid tissue in the large intestine of bactrian camels (Camelus bactrianus). Anat Rec (Hoboken).

[14] Doughri AM, Altera KP, Kainer RA (1972). Some developmental aspects of the bovine fetal gut. Zentralbl Veterinarmed A.

[15] Carlens O (1928). Studien über das lymphatische gewebe des darmkanals bei einigen Haustieren, mit besonderer Berücksichtigung der embryonalen Entwicklung, der Mengenverhältnisse und der Altersinvolution dieses Gewebes im Dünndarm des Rindes. Brain Struct Function.

[16] Reynolds JD, Morris B (1983). The evolution and involution of Peyer’s patches in fetal and postnatal sheep. Eur J Immunol.

[17] Pabst R, Geist M, Rothkotter HJ, Fritz FJ (1988). Postnatal development and lymphocyte production of jejunal and ileal Peyer’s patches in normal and gnotobiotic pigs. Immunology.

[18] Wang WH (2003). Observations on aggregated lymphoid nodules in the cardiac glandular areas of the bactrian camel (Camelus bactrianus). Vet J.

[19] Xu XH, Wang WH, Gao Q, Qi SS, He WH, Tai LF (2010). The anatomical characteristics of the aggregated lymphoid nodule area in the stomach of bactrian camels (Camelus bactrianus) of different ages. Vet J.

[20] Jia Lu Yu-jiao, Cheng Xiao-hong, Xu et al. Developmental characteristics of aggregated lymphoid nodules area in abomasum of fetal camels (Camelus bactrianus), 12 July 2023, PREPRINT (Version 1) available at Research Square [10.21203/rs.3.rs-3115891/v1]. 10.1186/s12917-024-04000-3 PMC11044426 38664826

[21] Zhang W-D, Zhang X-F, Cheng C-C, Jia S, Liu L, Wang W-H (2017). Impact of aging on distribution of IgA + and IgG + cells in aggregated lymphoid nodules area in abomasum of bactrian camels (Camelus bactrianus). Exp Gerontol.

[22] Hassan Omer ZI, Lu J, Cheng YJ, Li PX, Chen ZH, Wang WH (2023). Age-dependent changes in the anatomical and histological characteristics of the aggregated lymphoid nodules in the stomach of dromedary camels (Camelus Dromedarius). PLoS ONE.

[23] UniProt C (2021). UniProt: the universal protein knowledgebase in 2021. Nucleic Acids Res.

[24] Vicente-Manzanares M, Ma X, Adelstein RS, Horwitz AR (2009). Non-muscle myosin II takes centre stage in cell adhesion and migration. Nat Rev Mol Cell Biol.

[25] Lorenz M, Holmes KC (2010). The actin-myosin interface. Proc Natl Acad Sci U S A.

[26] Cooper GM. The Cell: A Molecular Approach. 2nd edition ed. Sunderland (MA): Sinauer Associates; 2000.

[27] Guo YJ, Pan WW, Liu SB, Shen ZF, Xu Y, Hu LL (2020). ERK/MAPK signalling pathway and tumorigenesis. Exp Ther Med.

[28] Vellend M, Cornwell WK, Magnuson-Ford K, Mooers AO, editors. Measuring phylogenetic biodiversity2010.

[29] Bargmann CI (1997). Olfactory receptors, vomeronasal receptors, and the organization of olfactory information. Cell.

[30] de Bock CE, Hughes MR, Snyder K, Alley S, Sadeqzadeh E, Dun MD (2017). Protein interaction screening identifies SH3RF1 as a new regulator of FAT1 protein levels. FEBS Lett.

[31] Mariman EC, Szklarczyk R, Bouwman FG, Aller EE, van Baak MA, Wang P (2015). Olfactory receptor genes cooperate with protocadherin genes in human extreme obesity. Genes Nutr.

[32] Muckenthaler M, Gray NK, Hentze MW (1998). IRP-1 binding to ferritin mRNA prevents the recruitment of the small ribosomal subunit by the cap-binding complex eIF4F. Mol Cell.

[33] Li K, Underhill DM (2020). C-Type lectin receptors in phagocytosis. Curr Top Microbiol Immunol.

[34] Geijtenbeek TB, Gringhuis SI (2016). C-type lectin receptors in the control of T helper cell differentiation. Nat Rev Immunol.

[35] van Vliet SJ, Garcia-Vallejo JJ, van Kooyk Y (2008). Dendritic cells and C-type lectin receptors: coupling innate to adaptive immune responses. Immunol Cell Biol.

[36] Tang C, Kamiya T, Liu Y, Kadoki M, Kakuta S, Oshima K (2015). Inhibition of Dectin-1 signaling ameliorates colitis by inducing Lactobacillus-mediated Regulatory T cell expansion in the intestine. Cell Host Microbe.

[37] Drummond RA, Dambuza IM, Vautier S, Taylor JA, Reid DM, Bain CC (2016). CD4(+) T-cell survival in the GI tract requires dectin-1 during fungal infection. Mucosal Immunol.

[38] Rochereau N, Drocourt D, Perouzel E, Pavot V, Redelinghuys P, Brown GD (2013). Dectin-1 is essential for reverse transcytosis of glycosylated SIgA-antigen complexes by intestinal M cells. PLoS Biol.

[39] Chiplunkar SV, Gogoi D (2019). The multifaceted role of Notch signal in regulating T cell fate. Immunol Lett.

[40] Brandstadter JD, Maillard I (2019). Notch signalling in T cell homeostasis and differentiation. Open Biol.

[41] Rocha BG-GD, Vassalli P (1995). Extrathymic T cell differentiation. Curr Opin Immunol.

[42] Olivares-Villagomez D, Van Kaer L (2018). Intestinal intraepithelial lymphocytes: sentinels of the Mucosal Barrier. Trends Immunol.

[43] Hageman JH, Heinz MC, van der Kretzschmar K, Clevers H, Snippert HJG (2020). Intestinal regeneration: regulation by the Microenvironment. Dev Cell.

[44] Koch U, Lehal R, Radtke F (2013). Stem cells living with a notch. Development.

[45] Morimoto RI (1993). Cells in stress: transcriptional activation of heat shock genes. Science.

[46] Rabindran SK, Giorgi G, Clos J, Wu C (1991). Molecular cloning and expression of a human heat shock factor, HSF1. Proc Natl Acad Sci U S A.

[47] Altieri DC, Stein GS, Lian JB, Languino LR (2012). TRAP-1, the mitochondrial Hsp90. Biochim Biophys Acta.

[48] Muranova LK, Shatov VM, Gusev NB (2022). Role of small heat shock proteins in the remodeling of actin microfilaments. Biochem (Mosc).

[49] Bakthisaran R, Akula KK, Tangirala R, Rao Ch M (2016). Phosphorylation of alphaB-crystallin: role in stress, aging and patho-physiological conditions. Biochim Biophys Acta.

[50] Collier RJ, Stiening CM, Pollard BC, VanBaale MJ, Baumgard LH, Gentry PC (2006). Use of gene expression microarrays for evaluating environmental stress tolerance at the cellular level in cattle. J Anim Sci.

[51] Hoter A, Amiri M, Warda M, Naim HY (2018). Molecular cloning, cellular expression and characterization of arabian camel (Camelus dromedarius) endoplasmin. Int J Biol Macromol.

[52] Warda M, Zeisig R (2000). Phospholipid- and fatty acid-composition in the erythrocyte membrane of the one-humped camel [Camelus dromedarius] and its influence on vesicle properties prepared from these lipids. Dtsch Tierarztl Wochenschr.

[53] Schopf FH, Biebl MM, Buchner J (2017). The HSP90 chaperone machinery. Nat Rev Mol Cell Biol.

[54] Zhang WD, Yao WL, He WH, Li JF, Wu XP, Chen ZH (2020). Bacterial community analysis on the different mucosal immune inductive sites of gastrointestinal tract in bactrian camels. PLoS ONE.

[55] McGhee JR, Fujihashi K (2012). Inside the mucosal immune system. PLoS Biol.

[56] Zhang WD, Wang WH, Li SX, Jia S, Zhang XF, Cao TT (2016). Localization of neonatal fc receptor for IgG in aggregated lymphoid nodules area in abomasum of bactrian camels (Camelus bactrianus) of different ages. BMC Vet Res.

[57] Tapley TL, Korner JL, Barge MT, Hupfeld J, Schauerte JA, Gafni A (2009). Structural plasticity of an acid-activated chaperone allows promiscuous substrate binding. Proc Natl Acad Sci U S A.

[58] Low WY, Rosen BD, Ren Y, Bickhart DM, To TH, Martin FJ (2022). Gaur genome reveals expansion of sperm odorant receptors in domesticated cattle. BMC Genomics.

[59] Cuklina J, Lee CH, Williams EG, Sajic T, Collins BC, Rodriguez Martinez M (2021). Diagnostics and correction of batch effects in large-scale proteomic studies: a tutorial. Mol Syst Biol.

[60] Vaudel M, Barsnes H, Berven FS, Sickmann A, Martens L (2011). SearchGUI: an open-source graphical user interface for simultaneous OMSSA and X!Tandem searches. Proteomics.

[61] Vaudel M, Burkhart JM, Zahedi RP, Oveland E, Berven FS, Sickmann A (2015). PeptideShaker enables reanalysis of MS-derived proteomics data sets. Nat Biotechnol.

[62] Reijnders M, Waterhouse RM (2021). Summary visualizations of gene ontology terms with GO-Figure!. Front Bioinform.

[63] Breuer K, Foroushani AK, Laird MR, Chen C, Sribnaia A, Lo R (2013). InnateDB: systems biology of innate immunity and beyond–recent updates and continuing curation. Nucleic Acids Res.

